# A Review of Personality-Targeted Interventions for Prevention of Substance Misuse and Related Harm in Community Samples of Adolescents

**DOI:** 10.3389/fpsyt.2018.00770

**Published:** 2019-01-22

**Authors:** Hanie Edalati, Patricia J. Conrod

**Affiliations:** Department of Psychiatry, CHU Sainte-Justine Research Center, University of Montreal, Montreal, QC, Canada

**Keywords:** school-based substance use prevention programme, community-based targeted prevention, cluster randomized trial, substance use outcomes, mental health, high-risk adolescents

## Abstract

Several school-based prevention programmes have been developed and used to prevent, delay, or reduce substance misuse, and related problems among community samples of adolescents. However, findings indicate that many of these interventions are associated with null, small, or mixed effects in reducing adolescent substance misuse, in particular for those mostly at risk of transitioning to substance use disorders. These findings highlight the need to shift the focus of substance use prevention efforts toward intervention strategies which directly target high-risk adolescents. The Preventure programme was designed to target four personality risk factors for substance misuse: hopelessness, anxiety sensitivity, impulsivity, and sensation seeking. This article reviews findings from the previous trials of personality-targeted interventions (i.e., Preventure programme) with adolescents and discuss the promises and benefits of these interventions for targeting community samples of high-risk adolescents at school level for reducing substance misuse and related mental health problems. Findings indicated that this programme has been successful in reducing the rates of alcohol and illicit drug use and substance-related harms by ~50% in high-risk adolescents with the effects last for up to 3 years. These interventions were also associated with a 25% reduction in likelihood of transitioning to mental health problems, such as anxiety, depression, suicidal ideation, and conduct problems. The programme is particularly beneficial for youth with more significant risk profiles, such as youth reporting clinically significant levels of externalizing problems, and victimized adolescents. A key strength of the Preventure programme is that it is embedded in the community and provides substance use intervention at school level to the general samples of high-risk adolescents who might not otherwise have access to those programmes.

## Introduction

Several school-based prevention programmes have been developed and used to prevent, delay, or reduce substance misuse and related problems among community samples of adolescents [e.g., (1–3)]. However, the majority of these interventions have not been evaluated using controlled randomized trials and do not meet the scientific standards against which many other medical interventions are compared (4, 5). A recent review of 46 systematic reviews on the effectiveness of interventions to prevent substance abuse among adolescents indicated that school-based prevention programmes were the most highly evaluated interventions for targeting adolescent substance abuse compared to other platforms including family/community-based interventions, digital platforms, policy interventions, multicomponent interventions, and incentives (6). Results from these reviews have indicated that many of these interventions are associated with null, small, or mixed effects with respect to reducing substance misuse among adolescents [e.g., (1–3, 7–9)]. There is also very limited evidence on medium or long-term impact of these interventions (7, 10), however, a small number of well-evaluated programmes do indeed indicated long-term benefits [e.g., (11)].

Among effective school-based prevention programmes, prevention programmes targeting never-smokers, and combined social competence and social influences curricula reduced smoking initiation (8). Smoke-Free Class Competition (SFC) decreased current smoking (12). School-based brief alcohol interventions (BAIs) were associated with reduced alcohol consumption (13). Combined social competence and social influence interventions were effective in preventing drugs and cannabis use (14, 15). Finally, programmes combining antidrug information with refusal skills, self-management skills, and social-skills training were linked to reduction in marijuana and alcohol use (16) [see (6)]. The majority of these intervention programmes are based on universal approaches which target all students, regardless of their level of risk for substance use and are based on delivering generic intervention components that are appropriate for general populations of adolescents [e.g., (17, 18)]. Therefore, adolescents who are most at risk of transitioning to substance use disorders, and those who have already started using substances may not benefit from these approaches (19). Some programmes were only successful when delivered individually and had no significant effect when delivered in groups (13) which requires many resources. Many programmes were effective when they incorporated components of multiple prevention models (8, 15, 16), included several intervention sessions (e.g., ≥ 15 sessions), or were facilitated by individuals other than teachers (15) which put a heavy burden on school systems. While programmes such as brief school-based interventions have been shown to reduce alcohol problem symptoms in young drinkers, they are limited in the extent to which they can address the full spectrum of drug-related behaviors and prevention aims (7). In addition, implementation fidelity is a significant challenge, with programs rarely being implemented in the way that they were originally designed and tested—due to limited resources and lack of mechanisms for promoting sustainability of prevention efforts [see Ennett et al. (20)]. One of the most widely studied school-based program was even shown to produce harmful effects when implemented through a program delivery system for which it was not initially intended (21). Despite these effects, substance use and misuse remain a highly prevalent problem and an escalating problem in certain high-risk communities and populations (22, 23).

The combination of limited efficacy or effectiveness of programmes and limited resources in the school settings to invest in prevention universally have stimulated research focusing on risk for early onset or more problematic substance use for the purpose of directing resources at those most in need of intervention, and to better understand and target risk trajectories to produce more impactful interventions (24). These findings suggest that shifting the focus of substance use prevention efforts toward more selective and targeted intervention strategies to target youth most at risk of transitioning to substance misuse and disorders might result in more impactful interventions, both from a cost-benefit perspective and potentially from an efficacy perspective. The distinction between selective and targeted programmes is of importance, where the former would simply direct existing interventions toward those most in need, and the latter would involve intervening upon the specific risk factor in a particular high-risk group [e.g., (25, 26)]. A combination of the selective and targeted approach is not always possible due to inability to target some known risk factors (e.g., gender), but when selective programs have been developed to directly target factors that render youth at greater risk for problematic use, such programmes have been shown to be highly beneficial for adolescents with higher risk profiles, both at individual (e.g., high-risk personality profiles) and contextual/environmental (e.g., poverty, trauma) levels (25–29).

One selective and targeted programme that has been widely tested in recent years is the “Preventure” programme, which targets personality risk factors through brief, selective interventions for groups of youth reporting higher levels of traits indicating risk. This personality-targeted approach to substance use prevention offers many advantages over more traditional universal prevention approaches by allowing schools and communities to direct their limited resources to those most in need, and by providing an integrated framework for addressing multiple prevention targets (e.g., substance use, mental health, victimization). A previous review article of personality-targeted interventions (27) included results from both adolescents and community and clinical adult samples and reported on delivery of the programme in any format, including full Preventure programme and personality-specific interventions (e.g., anxiety-sensitivity intervention for adults with high levels of anxiety sensitivity) and platform (e.g., delivered at school, delivered by telephone or email) (27). This article was focused on primary findings of each trial related to the substance use outcomes and does not include the secondary data analyses of these trials and outcomes related to mental health problems and other relevant outcomes (e.g., victimization). The present article reviews the findings from the five previous trials of personality-targeted interventions with community samples of at-risk adolescents delivered using school platform and discusses the promises of this approach for targeting early substance use and other mental health problems.

## Preventure Programme: A Personality-Targeted Approach to Substance Use Prevention

The Preventure programme was designed to target four personality risk factors for substance misuse: hopelessness, anxiety sensitivity, impulsivity, and sensation seeking (27). Longitudinal cohorts have identified these personality factors as strong and reliable predictors of future risk for substance use and related problems (30–32). These traits appear to be related to distinct substance use trajectories, differentiated on the basis of substance use and co-morbidity patterns, age of onset, and motivations for use [for a review, see (24)]. Sensation seeking trait is associated with biological and subjective sensitivity to the incentive rewarding and enhancing effects of substances and directly linked to early onset experimentation with substances and binge drinking, whereas, impulsivity, reflected as poor inhibition and a tendency to behave without proper consideration of the consequences, seems indirectly related to substance use through conduct problems, and therefore is associated with a slightly later onset of use, but more severe and problematic substance use profile (24, 33). By contrast, internalizing traits like hopelessness (i.e., a tendency to negative and depressive thinking) and anxiety sensitivity (i.e., a fear of anxiety-related physical sensations) are associated with the tendency to report using substances to cope and regulate negative affect and indirectly related to substance use through depressive and anxiety symptoms (24, 33). These traits are also able to predict specific drug-use profiles suggesting different underlying motivational drivers of the substance use, a hypothesis which has been confirmed by studies on the relationship between these traits and self-report motives for substance use in the general population (34), and in high-risk communities (35, 36).

Preventure is a “selective” substance use prevention programme, that is, high-risk students are selected based on their score on personality questionnaire, Substance Use Risk Profile Scale [SURPS (37)]. Adolescents who score one standard deviation above the population mean (e.g., school's mean) on one of the SURPS subscales (i.e., high-risk individuals; ~45% of the youth population) are invited to participate in brief group-based intervention sessions which target their dominant personality profile. Interventions are generally held during school hours and involve only two 90-min sessions, with 1 week separating sessions. Interventions are conducted using specific manuals for each personality profile that incorporate psycho-educational, motivational enhancement therapy (MET) and cognitive behavioral therapy (CBT) components and include real life “scenarios” shared by local youth with similar personality profiles [see (27)].

Personality-targeted interventions have been evaluated in eight randomized trials, including samples of adolescents and adults, in Canada, United Kingdom, Netherlands, and Australia, with additional trials in progress [for a review of primary findings of previous Preventure trials, see (27)]. Table 1 summarizes the findings from five previous randomized trials of personality-targeted interventions with community samples of high-risk adolescents. Findings from five previous trials with adolescent samples have indicated that the school-based Preventure Programme is successful in reducing a range of substance use outcomes by ~50% in high-risk adolescents with the effects lasting for up to 3 years [see (27)]. These interventions were also associated with a 25% reduced likelihood of transitioning to significant mental health problems, such as anxiety, depression, suicidal ideation, and conduct problems (38).

**Table 1:** Summary of five randomized trials of personality-targeted interventions (Preventure Programme) for substance misuse and related problems in community samples of high-risk adolescents.

**Trial**	**Sample**	**Substance use outcomes**	**Mental health outcomes**	**Other related outcomes**
1. Canadian preventure trial (4 months) (45)	HR secondary students (drinkers) IG: *n* = 166 CG: *n* = 131	Reduction in: Drinking rates (4 months) Drinking quantity (4 months) Binge drinking (4 months) Drinking problems (4 months)		
2. United Kingdom preventure trial (2 years) (40, 46–48)	HR secondary students IG: *n* = 190 CG: *n* = 157	Reduction in: Drinking rates (6 months) Binge drinking (6 months) Drinking problems (2 years) Uptake of illicit substance misuse (2 years) Drugs use rates (2 years) Drug use frequency (2 years) Cannabis use (2 years) Cocaine use (2 years)	Reduction in: Panic attack (6 months) Truancy (i.e., school avoidance) (6 months) Depression (6 months) Shoplifting (6 months)	
3. Dutch preventure trial (12 months) (44, 64)	HR secondary students (drinkers) IG: *n* = 343 CG: *n* = 356	Reduction in: Binge drinking (12 months) Growth of binge drinking (12 months)		Reduction in alcohol use outcomes in HR adolescents in lower education schools (e.g., vocational training)
4. United Kingdom adventure trial (2 years) (29, 39, 49, 51, 57, 59, 60, 64)	HR secondary students IG: *n* = 558 CG: *n* = 437	Reduction in: Drinking rates (2 years) Drinking quantity (2 years) Drinking frequency (2 years) Binge drinking (2 years) Growth of binge drinking (2 years) Drinking problems (2 years) Cannabis use (2 years)	Reduction in: Depressive symptoms (2 years) Anxiety symptoms (2 years) Conduct symptoms (2 years) Peer victimization (2 years) Bullying perpetration (2 years)	Reduction in alcohol use outcomes in HR adolescents with pre-existing depression and anxiety symptoms, and those in different SES (2 years)Additional reduction in those with pre-existing ADHD and conduct problems and those victimized by peers (6 months & 2 years)Reduction in drinking rates and growth of binge drinking in LR students (i.e., herd effect)
5. Australian CAP trial (3 years) (41)	HR secondary students IG: *n* = 202 CG: *n* = 291	Reduction in:Drinking rates (3 years) Binge drinking (3 years) Drinking problems (3 years)	

*Note. HR: High-risk; IG: intervention group; CG: control group; SES: Socioeconomic Status; LR: Low-risk; ADHD; attention-deficit/hyperactivity disorder; CAP: Climate and Preventure*.

## Facilitating Access to Care and Reducing the Barriers of Delivery for High-Risk Youth: A Community-Based Outreach Model to Substance Use Prevention

A key strength of the school-based Preventure programme is that it is embedded in the community. It provides preventative interventions to community samples of high-risk adolescents who might not otherwise have access to mental health programmes due to limited community resources, need to involve third party payers for such services, and due to local health care policies which often require that youth present with clinical impairment in order to receive services, or through their parents health insurance plan. To address these barriers, the Preventure programme is largely delivered by school personnel, including teachers and school counselors, but is designed to be adapted to the context in which youth present: trials indicate that Preventure is similarly efficacious when delivered by trained clinicians or school teachers in terms of reducing problematic mental health, alcohol and drug-related outcomes (39).

Results of the previous trials indicated that by targeting personality risk factors instead of onset of mental health or substance use problems, the programme also has the advantage of involving youth who might be higher functioning or not yet experiencing problems, allowing schools to promote it as a skill-building workshop and making it much more attractive and less intimidating to youth and their parents. Trials demonstrated that when the programme is promoted in this way, 70–85% of youth will voluntarily participate in the programme [see consort flow diagrams of trials (39–41)]. Delivering personality-specific skills in group format with adolescents with similar personality profiles may also help increasing engagement and empathy among adolescents and school personnel.

Finally, by training educational professionals to identify and intervene early on psychological risk factors for mental health and addiction, the programme equips professionals with assessment tools, and cognitive-behavioral and motivational interviewing skills that they can then use in future interventions with student who might require more intensive or additional services. In fact, a new trial in progress [i.e., Inter-Venture, (see https://ichgcp.net/clinical-trials-registry/NCT03114007)] delivers the 2-session Preventure programme in schools and then follows youth annually to then proactively identify and have a dialogue with high-risk youth about their persistent emotional and behavioral concerns or difficulties. These youth can then be rapidly identified and assisted (either by revisiting Preventure program in an individualized and extended version with the trained school counselor), or by being assisted in finding services or solutions to address their needs without having to wait for symptoms to escalate into crisis. This trial will evaluate whether integrating school-based prevention with community-based youth services is an effective method of reaching youth with mental health needs and preventing mental health problems at the population level.

The Preventure programme and its intervention materials are designed with consideration of cultural values, developmental needs, and attitudes of the targeted youth to make it more effective and relevant to adolescents receiving interventions in each personality group. O'Leary-Barrett et al. (40) have shown that youth-reported positive group experiences, learning, and skill development were predictive of positive behavioral changes in alcohol use and mental health symptoms after receiving the Preventure programme (42). Youth perspectives on the intervention independently accounted for up to 12–25% of the variance in changes in alcohol consumption and mental health symptoms over 12 months (42). These findings highlight the positive youth experiences of the programme as a significant indicator of its efficacy.

Adolescents may not be willing to share information regarding their substance use for fear of negative consequences and schools are often ambivalent about sharing drug-related information in the school context. Within the Preventure approach, substance use is not directly assessed or discussed. Participants are primarily selected based on their personality profiles. They learn about the target personality profile and associated risky coping behaviors, such as interpersonal dependence, aggression, avoidance, and substance misuse, using psycho-educational strategies. Thus, substance use is only discussed as one of the risky coping behaviors within a personality-focused learning context. Finally, the Preventure programme is very brief (only 2 sessions) and cost effective to implement. Because it does not interfere with the school curriculum, might prove to be easier to sustain compared to many universal approaches for youth substance use prevention [e.g., (43)].

## Effects on Substance Use Outcomes

Preventure targets personality traits that have been shown to associate with risk for early initiation of substance use and development of substance use disorders (24, 33). Thus, it can be helpful in the context of both prevention and early intervention for youth who have already started using substances. Previous trials which included substance use onset as an additional eligibility criterion have indicated that interventions were effective in reducing substance use and related problems in such groups of adolescents [e.g., (44, 45)].

Findings from all five previous Preventure trials with adolescents have reported a significant reduction with regard to a number of alcohol outcomes including drinking rates, drinking quantity, binge drinking, and problem drinking symptoms [up to 4 months; Canadian Preventure Trial; (45)], drinking rates and binge drinking (up to 6 months) and problem drinking symptoms (up to 2 years; United Kingdom (UK) Preventure Trial; (46–48), drinking rates, drinking quantity and frequency, rates and growth of binge drinking, and problem drinking symptoms [up to 2 years; Adventure Trial; (39, 49)], binge drinking and development of binge drinking [up to 12 months; Dutch Preventurec Trial; (44)], and drinking rates, binge drinking and alcohol-related harms [up to 3 years; Australian Climate and Preventure (CAP) Study; (41)]. Results from the U.K. Adventure and the Australian CAP trial also indicated a positive indirect effect of this intervention on the drinking rates and growth of binge drinking during the 24-month follow-up in the broader low-risk population of students (55% of age-matched school children) who were not selected for the intervention but were simply in the schools in which the Preventure programme was delivered to the high-risk students (i.e., “herd immunity”) (49).

Results from the UK Preventure trial also indicated that receiving interventions was associated with preventing uptake of illicit substance misuse and decreasing number of drugs used and drug use frequency in at-risk youth over a 2-year period (48). Importantly, the intervention increased the likelihood that high-risk adolescents survive as non-cannabis users by 30%, as non-cocaine users by 80%, and non-users of other illicit drugs by 50% over the 2-year follow-up period compared to high-risk youth in control group (48). Receiving interventions in the UK Adventure trial was also associated with significant reduction in rates of cannabis use at the 6-month follow-up and reductions in frequency of cannabis use at 12- and 18-month follow-up (50). With respect to specific personality profile, adolescents with high scores in sensation seeking were shown to be at higher risk for cannabis use and particularly benefited from the interventions with regard to delaying or reducing binge drinking (47) and delaying the onset of cannabis use (50). Youth in anxiety sensitivity group specifically benefited from the intervention in terms of showing fewer coping motives for drinking alcohol (46). Altogether, results across studies indicate a consistent moderate effect of Preventure on most substance use outcomes (27).

## Effects on Mental Health Problems and other Relevant Outcomes

There is a high comorbidity between substance misuse and a range of concurrent psychiatric disorders in adolescents (51). Specific personality traits have been identified as common underlying risk factors that explain the co-occurrence between substance misuse and psychiatric symptoms and disorders [e.g., (31)]. Preventure includes key components from CBT for major psychiatric disorders relevant to each type of the personality traits. This can be helpful in reducing psychiatric symptoms and improve mental health of adolescents receiving intervention (38). For example, CBT strategies for depression (52), are applied in the hopelessness intervention, CBT for panic disorder in the case of anxiety sensitivity (53, 54), and CBT for attention-deficit/hyperactivity disorder (ADHD) in the case of impulsivity (55) are integrated in the interventions and manuals used for each specific personality profile.

Secondary data analyses from previous trials have indicated that adolescents who received the Preventure programme improved on psychological problems including internalizing and externalizing outcomes. Results from the two-year follow-up trial with 1,024 adolescents (i.e., Adventure trial) indicated that receiving Preventure programme was associated with reduction in experiencing severe depression (26% reduction), anxiety (21% reduction), and conduct symptoms (21% reduction) over 2-year follow-up in the full sample (56). In addition, interventions significantly reduced the odds of severe depressive symptoms and conduct problems (56). An earlier trial similarly showed that interventions were associated with 18.2% reduction in self-report panic attacks and 15.3% in truancy (i.e., school avoidance) in anxiety sensitivity groups, reduction in depression scores in hopeless groups, and 8.5% reduction in shoplifting in the entire sample of 13–16 years old adolescents (*N* = 423, UK Preventure trial), with a stronger effect on this outcome for the impulsivity group (18% reduction in shoplifting) (40). Findings from the Australian CAP trial report very similar and longer-term benefits on mental health outcomes and suggest that these effects are specific to school-based interventions that target personality risk (57).

In addition to the positive effects on psychological outcomes, there is no indication that intervention effects are limited to the least problematic students at schools: adolescents with pre-existing mental health symptoms including depression, anxiety, ADHD, and conduct problem symptoms equally benefit (or receive more benefit) from Preventure with regard to their alcohol use outcomes. Secondary data analysis from the Adventure cluster-randomized trial (*n* = 3021) indicated that high-risk adolescents with depression and anxiety symptoms equally benefited from the interventions with regard to reduction in their alcohol use and related problems. Adolescents who reported higher symptoms of externalizing problems (ADHD and conduct problems) at baseline indicated more reduction in alcohol consumption at earlier follow-up periods (6 months post-intervention) and more reduction in their alcohol-related problems over the 24-month period compared to those with lower levels of these problems (58).

The programme has also been associated with some improvements in the rates of peer victimization and bullying perpetrations in schools receiving the interventions. Results from a study using the data from Adventure trial indicated that interventions were associated with reduction in peer victimization in the entire sample (Conrod et al., under review). In addition, there was a significant decrease in bullying perpetration among high-risk students in the intervention group, particularly among impulsive adolescents (Conrod et al., under review). This effect is particularly important given the high rates of co-occurrence between peer victimization and substance use during adolescence and the role of personality in susceptibility to victimization and perpetration of bullying (59, 60). These findings suggest that Preventure programme can provide an opportunity to deliver effective interventions for targeting adolescent substance use while also providing solutions for broader emotional and behavioral wellbeing in schools.

## Effects on Specific Groups of Adolescents

Most school-based intervention programmes have been evaluated at the community-level impact, whereas, the effect of contextual risk factors is also an important consideration in the evaluation of these programmes. Since contextual risk factors, such as socioeconomic status and peer victimization, increase the risk of early alcohol and substance misuse among adolescents [e.g., (60, 61)], it is probable that they also influence the effectiveness of substance use prevention programmes. Two recent studies involving secondary analyses of Preventure trials reported that the programme is particularly effective for youth with more significant risk profiles (29, 62).

A trial of Preventure in the Netherlands examined the effects of this programme on alcohol outcomes in 699 adolescents aged 13–15 years with different education levels within the Dutch school system (62). This study found that receiving Preventure reduced binge drinking, binge drinking frequency, alcohol use and alcohol use frequency in lower educated young adolescents (e.g., vocational training), but not in the higher education group (e.g., pre-university education) (62). Another study sought to investigate the potential moderating effects of socioeconomic status and peer victimization on the effectiveness of the Preventure programme in reducing adolescent alcohol use over a 2-year period using the data from Adventure cluster-randomized trial (*N* = 3021). Findings indicated that Preventure programme was equally beneficial for high-risk adolescents in different socioeconomic status and those exposed to peer victimization in terms of their alcohol outcomes and related problems (29). Receiving interventions was additionally beneficial for adolescents reporting peer victimization regarding their alcohol-related harm compared to non-victimized youth (29). Given previous findings linking peer victimization to risk for alcohol misuse and the tendency to report risky coping motives (e.g., to cope with negative emotions and to conform) (63), implementing the school-based Preventure programme provides an opportunity to deliver effective substance use interventions for victimized adolescents, who are at risk of long-term mental health concerns and substance misuse (64).

While Preventure is primarily designed to target the risk of substance use and related problems within the general populations of high-risk adolescents, it can be modified and adapted for use with youth populations with more complex or more specific needs. We recently launched a pilot project with high-risk adolescents in child welfare services to adapt Preventure for youth with experiences of trauma and maltreatment. In a recent manuscript, we reviewed *the available interventions for reducing substance use problems in adolescents involved in the child welfare system* and discussed the promises of personality-targeted interventions for reducing substance use problems in these populations (28). This review suggested that the Preventure programme is potentially a valuable targeted intervention for reducing high risk for substance use and mental health problems in adolescents involved in child welfare services, and for filling the gap in service delivery for these vulnerable populations (28).

## Limitations and Future Directions

Preventure programme has been evaluated in five trials with high-risk youth using school platform in different countries (40, 41, 44, 45, 49). Findings from these trials indicated a fairly robust effect on reduction of alcohol use outcomes. However, research on the efficacy of this approach on other types of substances is still limited and needs further investigations. For example, although results from previous trials have showed a significant reduction in likelihood of uptake and use of illicit substance misuse (48, 50), none of the previous trials reported any outcome related to smoking behavior or prescription drug use. Recent and ongoing trials of Preventure seek to address this gap. Two ongoing trials (Co-Venture and Inter-Venture) assess smoking behaviors. In addition, a new project, Canadian Underage Substance use Prevention (CUSP) trial (Conrod et al., 2018–2022), involving a total of 12,150 students in secondary schools across Canada, will investigate the effectiveness of the Preventure programme when delivered through a train-the-trainers model on illicit substance use in high-risk adolescents with prescription drug misuse as a novel secondary outcome. In addition to investigating different types of substances, personality-targeted interventions have yet to be tested in populations with higher proportion of high-risk adolescents, such as adolescents with histories of trauma or those reporting concurrent mental health problems. Our new project to adapt Preventure for youth with experiences of trauma and maltreatment in child welfare services, and the Inter-venture trial, which seeks to proactively identify and assist youth who are at higher risk of transition to significant psychiatric symptoms, are designed to address this gap. The impact of interventions on youth with special education needs or those with mental disabilities still needs an investigation. Moreover, there is a need to examine the effect of these interventions on other related problematic behaviors, such as risky driving and sexual behaviors, eating behaviors and risk for psychotic disorders. Finally, some studies, such as the CAP trial, have evaluated that programme against an evidence-based universal programme and showed equivalent effects on drinking behavior and slightly superior effects on higher risk outcomes, such as drinking problems (65). While this constitutes one form of control comparison, staunch methodologists might require additional research comparing the outcomes of the personality-targeted approach with a placebo control intervention delivered to high risk youth. While this might add to the quality of the evidence of this intervention approach, iatrogenic effects of psychosocial interventions with high risk youth have been reported in the literature [e.g., (66, 67)]. Therefore, any future attempt at placebo-controlled intervention designs should be very carefully considered.

## Conclusions

Findings from this article point to the importance of interventions which target modifiable risk factors associated with higher risk of initiation and development of substance use disorders, such as personality factors. Preventure programme is an evidence-based programme which has shown to be effective in reducing the risk for underage alcohol and illicit drug use, substance use related harms, and risk of transitioning to significant mental health problems in adolescence, with results last for up to 3 years. Additional efforts should be made to make school-based targeted interventions more accessible for high-risk youth in communities with insufficient resources to improve substance use and mental health outcomes.

## Author Contributions

HE and PC designed the concept of the Mini Review. HE wrote the first draft. PC edited for intellectual content.

### Conflict of Interest Statement

The authors declare that the research was conducted in the absence of any commercial or financial relationships that could be construed as a potential conflict of interest.
